# Nitric Oxide Synthase Inhibition Induces Renal Medullary Hypoxia in Conscious Rats

**DOI:** 10.1161/JAHA.118.009501

**Published:** 2018-07-24

**Authors:** Tonja W. Emans, Ben J. Janssen, Jaap A. Joles, C.T. Paul Krediet

**Affiliations:** ^1^ Internal Medicine‐Nephrology Amsterdam UMC / Academic Medical Centre at the University of Amsterdam The Netherlands; ^2^ Nephrology and Hypertension University Medical Centre Utrecht The Netherlands; ^3^ Pharmacology and Toxicology Maastricht University The Netherlands

**Keywords:** hypoxia, nitric oxide synthase, oxygen consumption, renal oxygenation, telemetry, Nephrology and Kidney, Hemodynamics, Hypertension, Blood Pressure

## Abstract

**Background:**

Renal hypoxia, implicated as crucial factor in onset and progression of chronic kidney disease, may be attributed to reduced nitric oxide because nitric oxide dilates vasculature and inhibits mitochondrial oxygen consumption. We hypothesized that chronic nitric oxide synthase inhibition would induce renal hypoxia.

**Methods and Results:**

Oxygen‐sensitive electrodes, attached to telemeters, were implanted in either renal cortex (n=6) or medulla (n=7) in rats. After recovery and stabilization, baseline oxygenation (pO_2_) was recorded for 1 week. To inhibit nitric oxide synthase, N‐ω‐nitro‐l‐arginine (L‐NNA; 40 mg/kg/day) was administered via drinking water for 2 weeks. A separate group (n=8), instrumented with blood pressure telemeters, followed the same protocol. L‐NNA rapidly induced hypertension (165±6 versus 108±3 mm Hg; *P*<0.001) and proteinuria (79±12 versus 17±2 mg/day; *P*<0.001). Cortical pO_2_, after initially dipping, returned to baseline and then increased. Medullary pO_2_ decreased progressively (up to −19±6% versus baseline; *P*<0.05). After 14 days of L‐NNA, amplitude of diurnal medullary pO_2_ was decreased (3.7 [2.2–5.3] versus 7.9 [7.5–8.4]; *P*<0.01), whereas amplitudes of blood pressure and cortical pO_2_ were unaltered. Terminal glomerular filtration rate (1374±74 versus 2098±122 μL/min), renal blood flow (5014±336 versus 9966±905 μL/min), and sodium reabsorption efficiency (13.0±0.8 versus 22.8±1.7 μmol/μmol) decreased (all *P*<0.001).

**Conclusions:**

For the first time, we show temporal development of renal cortical and medullary oxygenation during chronic nitric oxide synthase inhibition in unrestrained conscious rats. Whereas cortical pO_2_ shows transient changes, medullary pO_2_ decreased progressively. Chronic L‐NNA leads to decreased renal perfusion and sodium reabsorption efficiency, resulting in progressive medullary hypoxia, suggesting that juxtamedullary nephrons are potentially vulnerable to prolonged nitric oxide depletion.

**Clinical Perspective**

**What Is New?**

- This study shows, for the first time, the temporal development of renal cortical and medullary oxygenation during chronic nitric oxide (NO) synthase inhibition in unrestrained conscious rats.
- Chronic (2 weeks) NO synthase inhibition increased blood pressure and was associated with a progressive decline in renal medullary oxygenation levels whereas cortical oxygenation levels, after a transient decrease, returned to baseline.
- Chronic NO synthase inhibition attenuated the circadian fluctuations in medullary oxygenation.

**What Are the Clinical Implications?**

- Extrapolation of these experimental data strengthen the view that renal hypoxia is a causative factor in induced chronic renal damage, especially under conditions when medullary NO availability is limited.
- Interventions that prevent renal medullary hypoxia and increase NO production might protect against the development of chronic kidney disease.

## Introduction

Renal hypoxia is thought to be a crucial factor in the vicious circle of disease progression leading to kidney failure. In the past 2 decades, many studies have unraveled parts of the pathological role of chronic renal hypoxia during onset and disease progression. However, information regarding location (cortex or medulla) and time course of renal hypoxia is as yet incomplete,^1^ as is the role of established vasoactive substances. To fully understand the role of hypoxia in kidney failure, we need to locate and follow changes in renal oxygenation (pO_2_) over time. For this justification, the main goal of this study was to continuously record dynamics in renal pO_2_ in both cortex and medulla in a model known for the development of kidney injury. Previously, we measured renal tissue pO_2_ in the rat kidney during angiotensin‐II–induced hypertension and found a surprising contrast between acute and chronic effects.^2^, ^3^ Here, we measured oxygenation over time during deprivation of nitric oxide (NO) by NO synthase (NOS) inhibition.

The actions of NO have a strong link to oxygenation.^4^, ^5^ On the one hand, NOS inhibition decreases renal blood flow,^4^ thus oxygen delivery. In particular, NO is responsible for medullary flow.^6^ The majority of renal NOS activity is located in the medulla.^7^ On the other hand, NOS inhibition also increases renal oxygen consumption.^8^ Oxygen consumption efficiency is decreased in diabetic rats because of disturbed NO metabolism.^9^ Furthermore, NO modulates pressure natriuresis and^10^ tubuloglomerular feedback,^11^ and parenchymal NO bioavailability depends on tissue oxygenation in the kidney.^12^ Consequently, it is not surprising that, in the clinic as well as in animal models, NO deficiency is causally related to kidney disease. NOS inhibition leads dose dependently to severe hypertension, proteinuria, glomerulosclerosis, and reduced glomerular filtration rate (GFR) and renal plasma flow (RPF).^13^, ^14^ In patients with diabetic nephropathy, NO generation is limited by endothelial dysfunction.^15^ Also, in patients with chronic kidney disease, NO production is significantly lower.^16^, ^17^ Strategies to restore NO bioavailability appear beneficial in animals^18^, ^19^ and humans.^20^, ^21^

Various experimental animal models with l‐arginine analogues to induce renal vasoconstriction and hypertension have been used.^22^ Brezis et al induced NOS inhibition by L‐NG‐monomethylarginine in anesthetized rats, while measuring renal pO_2_ with Clark‐type microelectrodes. They showed that NOS inhibition acutely reduced pO_2_ in the renal medulla, but not in the cortex.^23^ Long‐term N(ω)‐nitro‐l‐arginine methyl ester (L‐NAME) treatment reduced oxygenation in kidneys of healthy and diabetic rats as measured by blood oxygen level–dependent magnetic resonance imaging.^24^ Also, in humans, acute administration of l‐arginine analogues was associated with blood oxygen level–dependent magnetic resonance imaging detectable reductions in renal pO_2_ that were most prominent in the medulla.^25^ These studies point to a preferential reduction of pO_2_ in the renal medulla during acute NOS inhibition. However, these measurements were obtained on a single time point, and chronic and/or transient changes in renal oxygenation could not be established.

Therefore, the current study was designed to assess the temporal effects of chronic NOS inhibition in the renal cortex and medulla. We used N‐ω‐nitro‐l‐arginine (L‐NNA) to induce renal vasoconstriction and hypertension in conscious rats, as previously done at our laboratory,^26^ and measured continuously blood pressure and renal cortical and medullary pO_2_ by telemetry. We hypothesized that changes in renal pO_2_ would occur early in the disease process and precede the development of severe renal damage in this model. For that reason, measurements were done during normal conditions first and then during 2 weeks of L‐NNA treatment and not in the phase when renal damage is established (>4 weeks). In addition, we analyzed the circadian rhythmicity of pO_2_ in the 2 conditions (control and L‐NNA). Healthy renal pO_2_ in cortex and medulla shows circadian rhythmicity.^27^ NO depletion is known to affect the diurnal rhythm of blood pressure in rodents,^28^, ^29^, ^30^ but effects on renal oxygenation circadian rhythm are as yet unknown.

## Methods

The data, analytical methods, and study materials will be made available on request to other researchers for purposes of reproducing the results or replicating the procedure.

### Animals

Male Sprague–Dawley rats (Envigo, Venray, The Netherlands), weighing 300 to 350 g, were cohoused in pairs. Animals were kept on a 12‐hour light/dark cycle with lights on at 6:00 am (Zeitgeber Time [ZT] 0), and lights off at 6:00 pm (ZT 12) and allowed access to water and standard rat chow ad libitum. Sensors were implanted in rats, as described below, to measure renal oxygenation in cortex (n=6) and medulla (n=7) and to measure blood pressure (n=8). Rats without sensors and treatment (n=7) were used to evaluate control renal function. All procedures were approved by the Animal Ethics Committee of Utrecht (DEC nr. 2014.II.03.015) and were in accord with the Dutch Codes of Practice for the Care and Use of Animals for Scientific Purposes.

### System Overview

Oxygen‐sensitive carbon paste electrodes were attached to telemeters (TR57Y; Kaha Sciences Ltd, Auckland, New Zealand) and calibrated for linearity before implantation.^31^ In short, surgery was performed under anesthesia, induced with 5% isoflurane, and maintained at 2% to 3% added to 100% O_2_. Electrodes were implanted in the right kidney as described in detail.^32^ The electrode tip was placed in the cortex or medulla, at appropriate depths (0.8–1.2 or 3.5–4.0 mm, respectively). Depth of electrodes was verified at the end of the experiment. The telemeter, including the battery, was placed in the abdominal cavity, sutured on the abdominal muscle. After surgery, rats were kept on a warm pad overnight to facilitate recovery. Activation of the telemetric pO_2_ measurements was achieved by placing the rat's cage on a Smartpad (TR181; Kaha Sciences).

Mean arterial pressure (MAP) and heart rate were recorded by telemetry (TRM54P; Kaha Sciences) in other rats. Pressure catheters were placed in the abdominal aorta just above the bifurcation of the common iliac arteries using similar anesthetic procedures. After 24 hours of recovery, the animal cage was placed on an external receiver pad (TR181; Kaha Sciences) to start recording of MAP and heart rate. All rats recovered for at least 1 week.

### NOS Inhibition and Urine Collection

After at least 1 week of baseline recording, NOS was inhibited by N‐ω‐nitro‐l‐arginine (L‐NNA; Sigma‐Aldrich, Zwijndrecht, The Netherlands) added to drinking water (40 mg/kg/day)^26^ for 2 weeks. Urine was collected by placing rats in metabolic cages for 24 hours, both during the baseline recording and after 2 weeks of NOS inhibition. Urine was collected from ZT 12 to 0 (dark phase) and ZT 0 to 12 (light phase). Note that creatinine clearance was calculated using the plasma creatinine concentration at the beginning of the terminal experiment.

### Assessment of Renal Function and Oxygen Use

Renal function was assessed in rats (n=21) after 2 weeks of NOS inhibition, as described before,^33^ and compared with values obtained in age‐matched controls (n=7). In short, rats were anesthetized with isoflurane and artificially ventilated (UNO, Zevenaar, The Netherlands). The femoral artery was cannulated for collection of plasma. Urine was collected through the bladder and left ureter. The jugular vein was cannulated for infusion of inulin, to calculate GFR, and of para‐aminohippuric acid, to calculate RPF. Renal blood flow (RBF) was calculated as RPF/1‐hematocrit. Blood gas variables in samples from the left renal vein and femoral artery were analyzed on a blood gas analyzer (GEM 4000; Instrumentation Laboratory, Bedford, MA). Kidney oxygen consumption (QO_2_, μmol/min) was calculated from the arteriovenous difference in oxygen content with a standard equation (oxygen content=[Hb]·O_2_ saturation·1.34+pO_2_·0.003) multiplied by RBF.^2^ At the end of the renal function measurements study, kidneys were collected, weighed, and processed for quantification of injury.

### Histology

Right kidneys were fixed for 24 hours in 10% formalin and embedded in paraffin. Slides (3 μm) were stained with periodic acid Schiff. For the L‐NNA group, 7 rats were chosen with proteinuria closest to the median value of the entire L‐NNA group (n=21). Kidney sections were scored for glomerulosclerosis and tubulo‐interstitial damage.^34^ Subcapsular‐ and juxtamedullary‐located glomeruli were analyzed separately. Analysis was performed by an experienced technician in a blinded manner. Glomerular endothelium was determined by JG12 immunohistochemistry. Results are presented as the percentage of JG12^+^ endothelial area in the glomerulus.^35^

### Statistical Analysis

**Figure 1:**
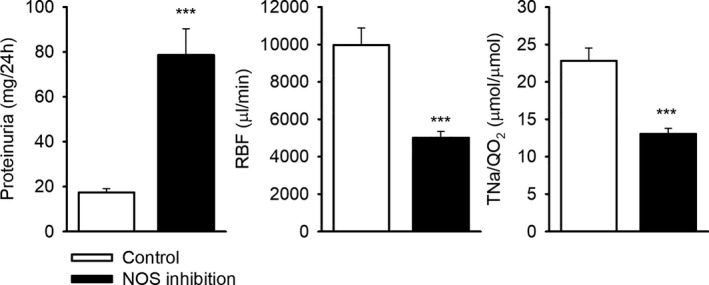
Proteinuria, renal blood flow (RBF), and sodium reabsorption efficiency (TNa/QO_2_) after 2 weeks of nitric oxide synthase (NOS) inhibition (n=21) compared with controls (n=7). Proteinuria was measured from urine collected in metabolic cages for 24 hours. Hemodynamics were measured under isoflurane anesthesia. ***P≤0.001 vs control, unpaired Student *t* test.

**Figure 2:**
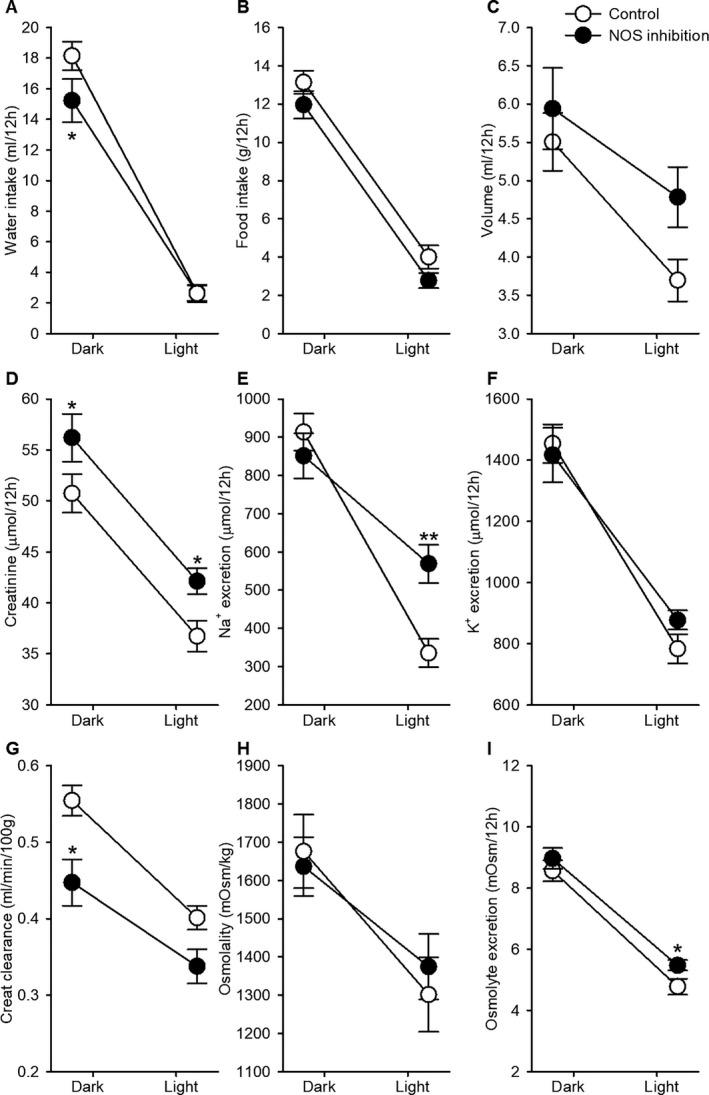
Dark‐ and light‐phase analysis of urine and water/food intake. Rats were individually housed in metabolic cages for 24 hours. Urine was collected during the dark‐phase (active) and during the lights‐phase (resting) period. Data are expressed as mean±SEM for (A) water intake, (B) food intake, (C) urine volume, (D) creatinine excretion, (E) Na^+^ excretion, (F) K^+^ excretion, (G) creatinine clearance, (H) urine osmolality, and (I) osmolyte excretion. *P<0.05; **P<0.001 vs control, ANOVA for repeated‐measures post hoc Tukey test. NOS indicates nitric oxide synthase.

**Figure 3:**
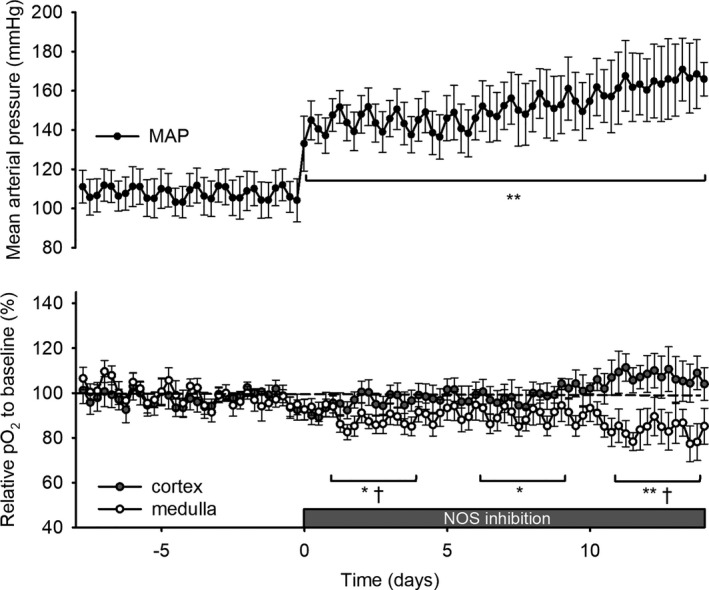
Cortical and medullary oxygenation (pO_2_) and mean arterial pressure (MAP) during 2 weeks of nitric oxide synthase (NOS) inhibition. N‐ω‐nitro‐l‐arginine (40 mg/kg/day) was added to drinking water on day 0 after at least 1 week of baseline measurements. Telemetric recordings of cortical (closed circles, n=6) and medullary (open circles, n=7) oxygenation (pO_2_) were recorded continuously. Values are expressed as a percentage of the baseline period before NOS inhibition. MAP was determined by telemetry (black dots, n=8) in another subset of animals. Data points represent the mean of 6‐hour averages±SEM. ANOVA for repeated measures with Tukey post hoc was performed: *P≤0.05; **P≤0.01 vs baseline in MAP or medulla; ^†^ *P*≤0.05 vs baseline in cortex.

**Figure 4:**
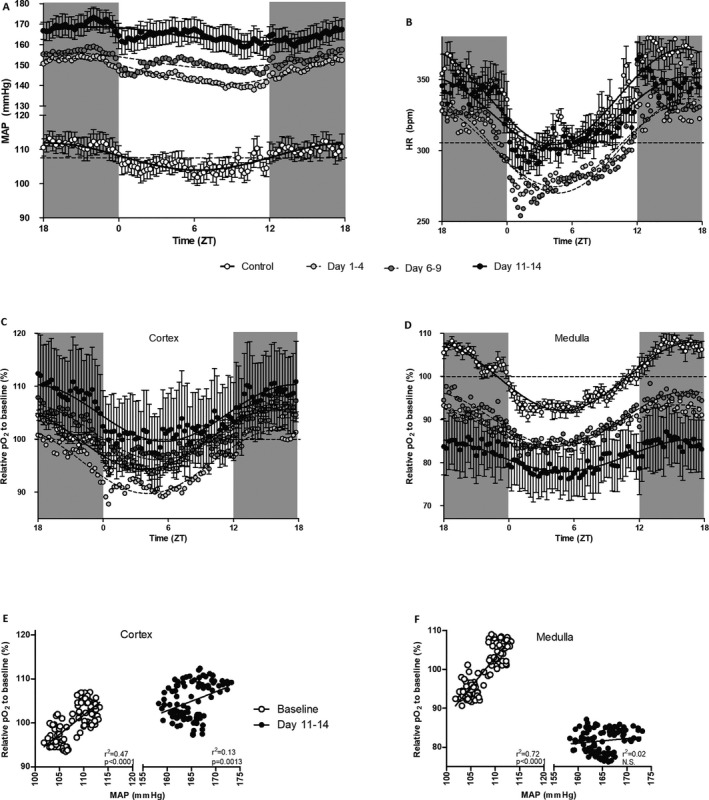
Circadian rhythmicity during nitric oxide synthase (NOS) inhibition in mean arterial pressure (MAP; A), heart rate (HR; B), cortical oxygenation (pO_2_; C), and medullary pO_2_ (D), and relation between MAP and pO_2_ in cortex (E) and medulla (F). All data are plotted as 15‐minutes mean values as recorded over 4 days in each rat, before (open circles, mean±SEM) and during 1 to 4 (light gray circles, mean only), 6 to 9 (dark gray circles, mean only), and 11 to 14 days of NOS inhibition (closed circles, mean±SEM). Dashed line represents circadian‐rhythm–adjusted mean before NOS inhibition. Data in (A through D) were analyzed by cosinor analysis (period=24 hours). ZT indicates Zeitgeber Time.

An unpaired Student *t* test was performed on terminal data (Figure 1), and a 2‐way ANOVA for repeated‐measures and post hoc Tukey test was performed on 12‐hour urine data (Figure 2). Original pO_2_ data were recorded in nA by carbon paste electrodes attached to oxygen telemeters at 4 Hz. The off‐set value was determined postmortem and subtracted from the original pO_2_ data. Baseline (nA) values were determined after recovery during 5 days before NOS inhibition and set at 100% (Figure 3). Artefacts were removed when the first‐order derivative exceeded a threshold of 5 nA/s, as described.^2^ Telemetry data, summated over 6 hours (Figure 3) and 15‐minute periods (Figure 4), are expressed as averages±SEM. One‐way ANOVA repeated measures with Tukey post hoc was performed on cortex and medulla data separately (Figure 3). Cosinor analysis, containing a period of 24 hours, was applied on telemetry data on day −4 to day −1, day 1 to day 4, day 6 to day 9, and day 11 to day 14 after NOS inhibition as described (Figure 4).^27^ Values derived from the Cosinor analysis, being MESOR (circadian‐rhythm–adjusted mean), amplitude, and acrophase (peak time), were compared together with those obtained in the control period. The derived cosine functions were compared using an extra‐sum‐of‐squares F test (GraphPad Prism version 5; Graphpad Software Inc, La Jolla, CA). Effects of MAP on pO_2_ (Figure 4E and 4F) were analyzed by pairing the mean pO_2_ and mean MAP data in matched periods of 15 minutes. During a defined baseline period (day −4 to day −1) and a standard experimental period (day 11 to day 14), we compared the regression lines of cortex and medulla with their own baseline using ANCOVA. The rationale for using the ANCOVA model is that it combines analysis of variance with linear regression. Mean pO_2_ is adjusted for the different levels of blood pressure. The ANCOVA model includes the squared deviates of pO_2_ and blood pressure, as well as the codeviates. A value of *P*<0.05 was considered significant.

## Results

### Effects of NOS Inhibition After 2 Weeks

To validate the efficacy of the 2‐week treatment of L‐NNA in drinking water, urine was collected by metabolic cages and hemodynamic parameters and renal tissue were collected during a terminal experiment under isoflurane anesthesia (Tables 1 and 2). NOS inhibition for 14 days resulted in proteinuria (78±12 versus 17±2 mg/day; *P*<0.01; Figure 1) and was associated with a reduction in GFR (1374±74 versus 2098±122 μL/min) and RPF (2801±181 versus 5861±497 μL/min) versus control (both *P*<0.001; Table 1). Calculated renal vascular resistance was increased (10.2±0.9 versus 30.5±2.5 mm Hg/mL/min; *P*<0.001). Total tubular sodium reabsorption (TNa) was proportionately reduced (182±10 versus 285±18 μmol/min; *P*<0.001), so that fractional TNa remained constant. Plasma creatinine concentration at the beginning of the terminal experiment was higher in L‐NNA–treated rats than in controls (53±3 versus 39±5 μmol/L; *P*<0.05).

**Table 1:** Renal Hemodynamics (Under Isoflurane Anesthesia) After 2 Weeks of NOS Inhibition in Rats

	Control	NOS Inhibition	*P* Value
N	7	21	
Final body weight, g	363±5	356±7	0.567
MAP, mm Hg	97±2	137±5	<0.001
GFR, μL/min	2098±121	1374±74	<0.001
RPF, μL/min	5861±497	2801±181	<0.001
RVR, mm Hg/mL/min	10.2±0.9	30.5±2.5	<0.001
FF, %	37±1	50±1	<0.001
TNa, μmol/min	285±18	182±10	<0.001
FTNa, %	13.6±0.2	13.2±0.1	0.134

Data are expressed as mean±SEM. FF indicates filtration fraction; FTNa, fractional tubular sodium reabsorption; GFR, glomerular filtration rate; MAP, mean arterial pressure; NOS, nitric oxide synthase; RPF, renal plasma flow; RVR, renal vascular resistance; TNa, tubular sodium reabsorption.

**Table 2:** Renal Injury Scores After 2 Weeks of NOS Inhibition in Rats

	Control	NOS Inhibition	*P* Value
N	7	7	
Proteinuria, mg/day	17±2	57±7	<0.01
Glomeruli			
Total glomerulosclerosis, %	0.7±0.3	6.6±1.3	<0.01
Subcapsular, %	0.6±0.4	8.6±0.7	<0.01
Juxtamedullar, %	0.8±0.5	4.6±2.4	0.149
Glomerular endothelium (JG12^+^ area/glomerulus [%])			
Subcapsular, %	13.4±1.3	11.0±1.6	0.240
Juxtamedullar, %	15.1±1.5	16.0±1.7	0.695
Tubulo‐interstitium infiltrate, score	0.20±0.06	0.58±0.10	<0.01
Fibrosis, score	0.34±0.07	0.94±0.14	<0.01
Atrophy, score	0.14±0.03	1.04±0.12	<0.001

Data are expressed as mean±SEM. NOS indicates nitric oxide synthase.

Two weeks of L‐NNA induced mild histological damage in both cortex and medulla (Table 2). Oxygen consumption efficiency measured by terminal sodium reabsorption efficiency (TNa/QO_2_) decreased (13.0±0.8 versus 22.8±1.7 μmol/μmol, *P*<0.001). Excretion of Na^+^, K^+^, and creatinine peaked during the dark‐phase (active phase) in both control and L‐NNA–treated rats. Na^+^ (569±50 versus 335±37 μmol/12 hours) and creatinine (42±1 versus 37±2 μmol/12 hours) excretions were increased during the light phase (resting phase) in L‐NNA–treated rats compared with controls (*P*<0.05). Creatinine clearance was decreased overall in L‐NNA–treated rats, but, as in control rats, still peaked during the dark phase (Figure 2).

### Temporal In Vivo Changes in pO_2_ and Blood Pressure

Measurements by telemetry showed that NOS inhibition rapidly induced systemic hypertension (165±6 versus 108±3 mm Hg; *P*<0.001; Figure 3). Telemetric pO_2_ recordings revealed that, after an initial dip during days 1 to 4 after NOS inhibition, cortical oxygenation returned to baseline and slightly increased after day 11 during NOS inhibition. In contrast, medullary pO_2_ decreased progressively during the full L‐NNA treatment (−19±6% during days 11 to 14 versus baseline; *P*<0.01; Figure 3). Circadian rhythmicity of MAP, recorded during days 11 to 14 of NOS inhibition, showed a phase shift to 4 hours later (22.3 [20.2–0.3] versus 18.3 [17.1–19.5] ZT). There was also a phase shift in cortical oxygenation to 2 hours later (17.6 [16.5–18.6] versus 15.8 [15.2–16.4) ZT]. Amplitude of heart rate decreased (26 [23–28] versus 34 [31–36] beats per minute; Table 3 and Figure 4) when compared with values obtained during baseline. Amplitude of the rhythm in medullary pO_2_ also decreased (3.7 [2.2–5.3] versus 7.9 [7.5–8.4] %; Table 3), but this was not the case in the cortex. MAP correlated to pO_2_ in cortex and medulla during baseline (both *P*<0.0001; Figure 4E and 4F). However, after 2 weeks of L‐NNA treatment, the correlation between MAP and pO_2_ was significantly diminished in the cortex (ANCOVA: *P*<0.01 versus baseline) and completely lost in the medulla (*P*<0.001 versus baseline).

**Table 3:** Circadian Parameters in Cortical and Medullary Oxygenation, MAP, and HR During NOS Inhibition

	Cortex (%) n=6	Medulla (%) n=7	MAP (mm Hg) n=8	HR (bpm) n=8
Mesor				
Con	100.0 [99.8–100.2]	100.0 [99.7–100.3]	107.9 [107.4–108.5]	338 [336–340]
Day 1 to 4	95.8 [95.0–96.6]^a^	88.7 [88.0–89.4]^a^	146.9 [146.3–147.5]^a^	304 [302–305]^a^
Day 6 to 9	101.3 [100.1–102.6]	90.2 [89.2–91.2]^a^	152.2 [151.4–153.1]^a^	303 [302–305]^a^
Day 11 to 14	104.9 [103.5–106.4]^a^	81.7 [80.6–82.8]^a^	165.1 [164.0–166.1]^a^	326 [324–328]^a^
Amplitude				
Con	5.7 [5.4–6.0]	7.9 [7.5–8.4]	4.0 [3.2–4.7]	34 [31–36]
Day 1 to 4	6.0 [4.9–7.2]	4.7 [3.7–5.7]^a^	6.3 [5.5–7.2]^a^	29 [27–31]
Day 6 to 9	5.3 [3.5–7.0]	6.4 [4.9–7.8]	3.7 [2.5–4.9]	33 [31–35]
Day 11 to 14	5.4 [3.4–7.3]	3.7 [2.2–5.3]^a^	3.6 [2.1–5.1]	26 [23–28]^a^
Acrophase (ZT hours)				
Con	15.8 [15.2–16.4]	16.9 [16.4–17.5]	18.3 [17.1–19.5]	16.3 [15.4–17.2]
Day 1 to 4	16.1 [15.2–17.1]	15.6 [14.7–16.5]	20.1 [19.2–21.0]	16.4 [15.4–17.4]
Day 6 to 9	17.1 [16.3–18.0]	16.5 [15.7–17.3]	20.1 [18.2–22.0]	16.7 [15.7–17.6]
Day 11 to 14	17.6 [16.5–18.6]^a^	17.0 [15.8–18.2]	22.3 [20.2–24.3]^a^	16.6 [15.3–17.9]

Data were analyzed by cosinor analysis (period=24 hours), lighting schedule; lights on at 6:00 am (Zeitgeber Time [ZT] 0), and lights off at 6:00 pm (ZT12). Mesor: circadian‐rhythm–adjusted mean; Acrophase: Peak time of cosine function (hours in ZT). All values shown as medians [95% CI]. CI indicates confidence interval; HR, heart rate; MAP, mean arterial pressure.

a *P*<0.05 vs control.

## Discussion

This study shows, for the first time, measurements that were done (1) in a continuous chronic setting showing the differential temporal changes in cortical and medullary oxygenation, (2) in the absence of confounding effects of anesthesia, and (3) over full 24‐hour periods allowing the analysis of circadian patterns during chronic NOS inhibition in unrestrained mammals (ie, rats). Cortical pO_2_ normalized and increased slightly during chronic L‐NNA treatment, whereas renal medullary pO_2_ decreased progressively during the 2‐week treatment period up to 19%. The observation that chronic NOS inhibition preferentially induces renal medullary hypoxia extends previous findings acquired in acute settings.^23^, ^25^

RBF was reduced in L‐NNA–treated rats and thus oxygen delivery. We measured this after 2 weeks; however, NO inhibition by l‐arginine analogues is known to acutely induce reductions in RBF.^9^, ^23^, ^25^ Reduced perfusion leads to reduced GFR and hence metabolic demand. Therefore, reduced RBF per se does not lead to reduced oxygen levels.^36^ However, the efficiency of sodium reabsorption was found to be decreased in NOS‐inhibited animals, as was also found in hypertensive^37^ and diabetic rats.^9^ In contrast, efficiency of sodium reabsorption was not (yet) significantly decreased in healthy animals during acute NOS inhibition.^9^ Therefore, we assume that the inefficiency of oxygen use played a role in the development of medullary hypoxia in this hypertensive model at long term, whereas RBF reduction caused acute hypoxia. In addition, most of the active metabolic demand is located in the outer medulla, where oxygen consumption efficiency is crucial.^36^

Differential in vivo effects of L‐NNA treatment on pO_2_ in the renal cortex and renal medulla cannot be directly linked to the changes in GFR and RBF and the decrease in sodium reabsorption efficiency, because in vivo effects of NOS inhibition on the various renal components are complex. The observed differences in oxygenation in the cortex and medulla raise the following questions: (1) Is there a differential effect of NOS inhibition on renal tubules?; (2) Is there a differential influence of NOS inhibition on blood flow distribution?; and (3) Can the vascular and/or tubular architecture influence the observations? All NOS isoforms are expressed in the kidney,^38^ and the synthesized NO itself is known to inhibit reabsorption of Na^+^ along the nephron by reducing the respiratory rate of mitochondria^8^, ^39^, ^40^, ^41^ therefore, NO inhibits oxygen consumption. Three lines of evidence support our observations.

First, isolated renal tubules derived from cortex or medulla do not show differences in sensitivity toward NO inhibition.^42^ However, NO‐mediated inhibition of respiration in renal tubular cells is particularly marked when oxygen levels are low.^42^ This implies that the effect of NO inhibition on tubular Na^+^ reabsorption is more effective in the relatively hypoxic medulla than in the well‐oxygenated cortex. Therefore, NOS inhibition has more impact on inducing hypoxia in tubular segments of the medulla than of the cortex. Besides, Deng et al showed that NOS inhibition decreased the efficiency of tubular respiration in isolated proximal tubules,^43^ whereas the proximal tubules are the most efficient tubules in Na^+^ reabsorption. NOS inhibition also reduced total reabsorption of Na^+^ in proximal tubules.^44^ On top of that, chronic NOS inhibition increased activity of Na‐K‐2Cl cotransporters in the thick ascending limbs of Henle's loop (TALH) along with the increase of blood pressure in rats.^45^ Together, these data suggest that NOS inhibition forced Na^+^ reabsorption from the proximal to medullary tubules, where Na^+^ is reabsorbed at higher oxygen costs. In line with our observations, NOS inhibition increases oxygen consumption, especially in the medulla.

Second, differential regional regulation of blood flow in the kidney has been measured during acute NOS inhibition in anesthetized^46^ and conscious animals^47^ and in humans.^48^ Vascular resistance in kidneys of L‐NAME–treated rats was greater in the medulla than in the cortex, which was caused by a shift of intrarenal blood flow and hence oxygen delivery from medulla to cortex.^49^ Arterioles of juxtamedullary glomeruli, which provide blood flow to the medulla, contain more smooth muscle than those of glomeruli in the cortex.^50^, ^51^ Consequently, blood supply to the medulla is more dependent on the NO vasodilatory reserve than the cortex.^52^ There is also substantial evidence that medullary flow, more than cortical flow, is associated with long‐term blood pressure control.^53^, ^54^ Intramedullary infusion of l‐arginine can restore systemic hypertension in salt‐sensitive rats on a high‐salt diet, presumably by preventing reductions in medullary blood flow.^55^ In line with our observations, NOS inhibition has more impact on oxygen delivery to the medulla than cortex.

Third, the architecture of the renal vasculature and tubules is arranged in such a way that the TALH are not very near their vascular oxygen supply, which makes the TALH prone to hypoxia in the first place. The countercurrent design of vasa recta maintains oxygen supply to the deep medulla, but also makes the TALH more vulnerable to hypoxia, as suggested by a detailed mathematical 3 dimensional model by Fry et al.^56^

Considering these differential responses of the renal cortex and medulla to NOS inhibition on tubular function, blood flow distribution, as well as architectural design, the findings in our study concur with the concept that chronic NOS inhibition leads to dissimilar changes in regional oxygenation.

Cortical and medullary pO_2_ were decreased during the acute phase of hypertension, wherein cortical oxygenation returned to baseline and medullary oxygenation seemed to stabilize at a lower level within the first week. We speculate that, during the acute phase, restricted oxygen delivery causes reductions in pO_2_ in cortex and medulla. Thereafter, reduced oxygen consumption efficiency causes a further reduction of pO_2_ in the medulla. Coincidentally, cortical oxygenation increases slightly and MAP increases progressively. These effects are probably blood‐pressure independent because (1) our data (Figure 4E and 4F) show that the direct relation between MAP and pO_2_ is depressed in the cortex and lost in the medulla after chronic NOS inhibition, and (2) previous findings show that renal alterations induced by NO deficiency cannot be restored by antihypertensive treatment.^57^ Welch et al showed that low renal oxygenation and inefficient oxygen consumption were not subject to hypertension in the spontaneously hypertensive rat.^58^

Diurnal patterns in renal oxygenation and electrolyte excretions were still intact during NOS inhibition in our study. However, the amplitude of medullary oxygenation was reduced. This suggests that NO causes diurnal fluctuations in medullary oxygenation. NO is involved in the regulation of circadian rhythmicity in blood pressure.^28^, ^29^, ^30^ A shift in peak blood pressure of 4 hours was found in the current study. This peak delay is probably not caused by the rhythm in L‐NNA intake, which occurs mainly during the dark‐active phase, because L‐NNA elimination is very slow (half‐life, 20 hours).^59^ In studies in mice, NO synthase inhibition had no effect on cardiovascular rhythms,^60^ but in rats a high dose of chronic L‐NAME treatment attenuated the amplitude of the blood pressure rhythm. The usual fall in blood pressure during the morning was less pronounced during high‐dose L‐NAME treatment. In the same study, chronic L‐NAME treatment caused a reduction in the day/night fluctuation in heart rate.^28^

Other studies have shown responses similar to ours in chronic and acute administration of NOS inhibition on renal hemodynamics.^61^ Unfortunately, in the absence of telemetric blood flow probes, we were limited to hemodynamic measurements at the end of the experiment. It is clear that development of hypoxia in the medulla has a progressive pattern and suggests an aggravation of the NOS inhibition model over time. However, we speculate that, at some point, renal damage should start to contribute to medullary hypoxia, rather than increasing vasoconstriction and decreasing oxygen consumption efficiency further over time. L‐NNA treatment for 2 weeks was accompanied with mild glomerulosclerosis. Knowing that chronic NOS inhibition progressively induces renal damage over time, we speculate that cortical oxygenation will also fall when L‐NNA treatment was continued over longer time frames. However, that was beyond the scope of the current study, which was focussed on the first 2 weeks in this model, before severe pathology developed, because we were interested whether changes in oxygenation could be a causative factor (and not a sequential result). Finally, this telemetry‐based oxygenation recording does not allow calibration into absolute values. We acquired a recording of a healthy baseline in every animal (cortex or medulla), and long‐term measurements were compared with baseline.

Cumulatively, this study proves the existence of medullary hypoxia upon chronic NOS inhibition in rats. This is attributed to constriction of renal vasculature, which was evident by reductions in GFR and RBF. Increased natriuresis did not balance renal oxygenation, because of decreased efficiency of oxygen consumption.

## Perspectives

Disturbance of renal oxygenation is a hallmark during development of kidney damage. Our results extend findings from acute experiments and confirm the association of hypoxia with the development of renal damage. Whether hypoxia, in this chronic model, is reversible, and whether this protects against hypertension and renal damage, is subject for further study.

## Sources of Funding

This work was supported by a ZonMw Clinical Fellowship (Krediet No. 40007039712461) and Dutch Kidney Foundation (Janssen Innovation call 16OI21).

## Disclosures

None.
